# MRI-derived features reveal recurrence heterogeneity in VI-RADS 2 non-muscle-invasive bladder cancer

**DOI:** 10.3389/fonc.2026.1921569

**Published:** 2026-09-03

**Authors:** Junpeng Sun, Ziyong Wang, Maofa Yang, Zijian Tan, Hong Sun, Xusong Pang, Longguo Dai, Siting Wang, Kun Zhang, Tenglu Ma, Zhao Cao, Hong Yang

**Affiliations:** Department of Urology I, The Third Affiliated Hospital of Kunming Medical University, Yunnan Cancer Hospital, Peking University Cancer Hospital Yunnan, Kunming, Yunnan, China

**Keywords:** bladder neoplasms, machine learning, magnetic resonance imaging, prognosis, recurrence

## Abstract

**Objectives:**

To characterize recurrence heterogeneity among patients with VI-RADS 2 non-muscle-invasive bladder cancer (NMIBC) and to develop and internally validate a recurrence-free survival (RFS) prediction model incorporating quantitative MRI features.

**Methods:**

This retrospective study included 342 patients with pathologically confirmed VI-RADS 2 NMIBC, who were randomly divided into training and internal validation cohorts. Clinical and quantitative MRI variables were evaluated using Cox regression, least absolute shrinkage and selection operator regression, Boruta, and recursive feature elimination. Six survival prediction algorithms were developed and compared. The selected model was further compared with a clinical Cox model and the European Association of Urology (EAU) risk stratification system using the concordance index (C-index), time-dependent area under the curve (AUC), calibration, Brier score, and decision curve analysis.

**Results:**

Among 342 patients, 111 (32.5%) experienced recurrence. Six predictors were retained: tumor number, maximum tumor vertical distance, maximum tumor diameter, maximum tumor-base contact length, mean apparent diffusion coefficient (ADC) at the tumor base, and minimum ADC within the tumor. The random survival forest (RSF) model achieved C-index values of 0.832 and 0.757 in the training and validation cohorts, respectively. In the validation cohort, the corresponding C-index values were 0.417 for the clinical Cox model and 0.534 for the EAU risk stratification system. The RSF model also yielded higher time-dependent AUCs than both reference models and greater net benefit at the 60-month horizon. RSF-derived risk groups showed significantly different RFS in both cohorts; in the validation cohort, the hazard ratio for the high-risk versus low-risk group was 5.30 (95% confidence interval, 2.39-11.76).

**Conclusions:**

Within this single-center cohort, recurrence risk varied substantially among patients with VI-RADS 2 NMIBC. An RSF model incorporating quantitative MRI features and tumor number showed better internal discrimination than the clinical Cox model and the EAU risk stratification system and separated patients into distinct risk groups. These findings suggest that quantitative MRI features may complement conventional clinical assessment in this imaging-defined subgroup. Prospective multicenter external validation with longer follow-up is required before the model can be considered for clinical use.

## 1 Introduction

Bladder cancer is one of the most common malignancies worldwide (1), and non-muscle-invasive bladder cancer (NMIBC) accounts for approximately 70% of newly diagnosed cases (2). Despite standard treatment with transurethral resection of bladder tumor (TURBT), often followed by intravesical therapy, NMIBC has a high recurrence rate, with 50%–70% of patients experiencing recurrence within 5 years (3). Accurate risk stratification is therefore important for risk-adapted surveillance and treatment planning.

The European Association of Urology (EAU) risk stratification system is widely used to estimate the risk of recurrence and progression in NMIBC (4). However, it is based primarily on clinicopathological variables, including tumor stage, grade, size, and number, and may not fully capture tumor heterogeneity. The Vesical Imaging Reporting and Data System (VI-RADS), derived from multiparametric magnetic resonance imaging (mpMRI), uses a five-point scale to estimate the likelihood of muscularis propria invasion and standardizes image interpretation (5, 6). VI-RADS has been validated for local staging (7), and higher VI-RADS scores have also been associated with a higher risk of recurrence (8). Kozikowski et al. further reported associations between VI-RADS scores of 3 or higher and adverse oncological outcomes, including early recurrence and progression (9).

A VI-RADS score of 2 indicates that muscularis propria invasion is unlikely; however, it does not directly characterize the recurrence risk after treatment for NMIBC. Quantitative imaging features, particularly diffusion-weighted imaging (DWI)-derived apparent diffusion coefficient (ADC) measurements, may reflect tumor microstructure and provide prognostic information beyond conventional clinical factors (10–12).

The prognostic value of MRI-derived quantitative features in patients with VI-RADS 2 lesions remains insufficiently studied (13). This study therefore aimed to characterize RFS in patients with VI-RADS 2 NMIBC and to develop and internally validate an MRI-based survival model integrating quantitative imaging features. We also compared its performance with a clinical Cox model and the EAU risk stratification system to explore the potential incremental value of imaging-based risk assessment in this subgroup.

## 2 Materials and methods

Detailed feature-selection results and model comparisons are provided in the **Supplementary Material**.

### 2.1 Study design and patient population

This retrospective study was approved by the institutional review board of our institution (approval no. KYLX2026-228). The requirement for written informed consent was waived because of the retrospective design. All procedures were conducted in accordance with the Declaration of Helsinki.

Consecutive patients with NMIBC who underwent treatment at our institution between January 2019 and December 2024 were screened for eligibility.

The inclusion criteria were as follows: (1) TURBT followed by a pathological diagnosis of urothelial carcinoma; (2) inclusion of muscularis propria in the surgical specimen and pathological confirmation of NMIBC; (3) availability of preoperative mpMRI performed within 4 weeks before surgery; (4) no radiotherapy or chemotherapy before MRI; and (5) a VI-RADS score of 2 for the target lesion, with no lesion scoring higher than 2 in patients with multiple tumors.

The exclusion criteria were as follows: (1) a history of another malignant tumor; (2) concurrent carcinoma *in situ*; (3) incomplete clinical or imaging data; (4) image quality insufficient for quantitative assessment; or (5) loss to follow-up.

**Figure 1:**
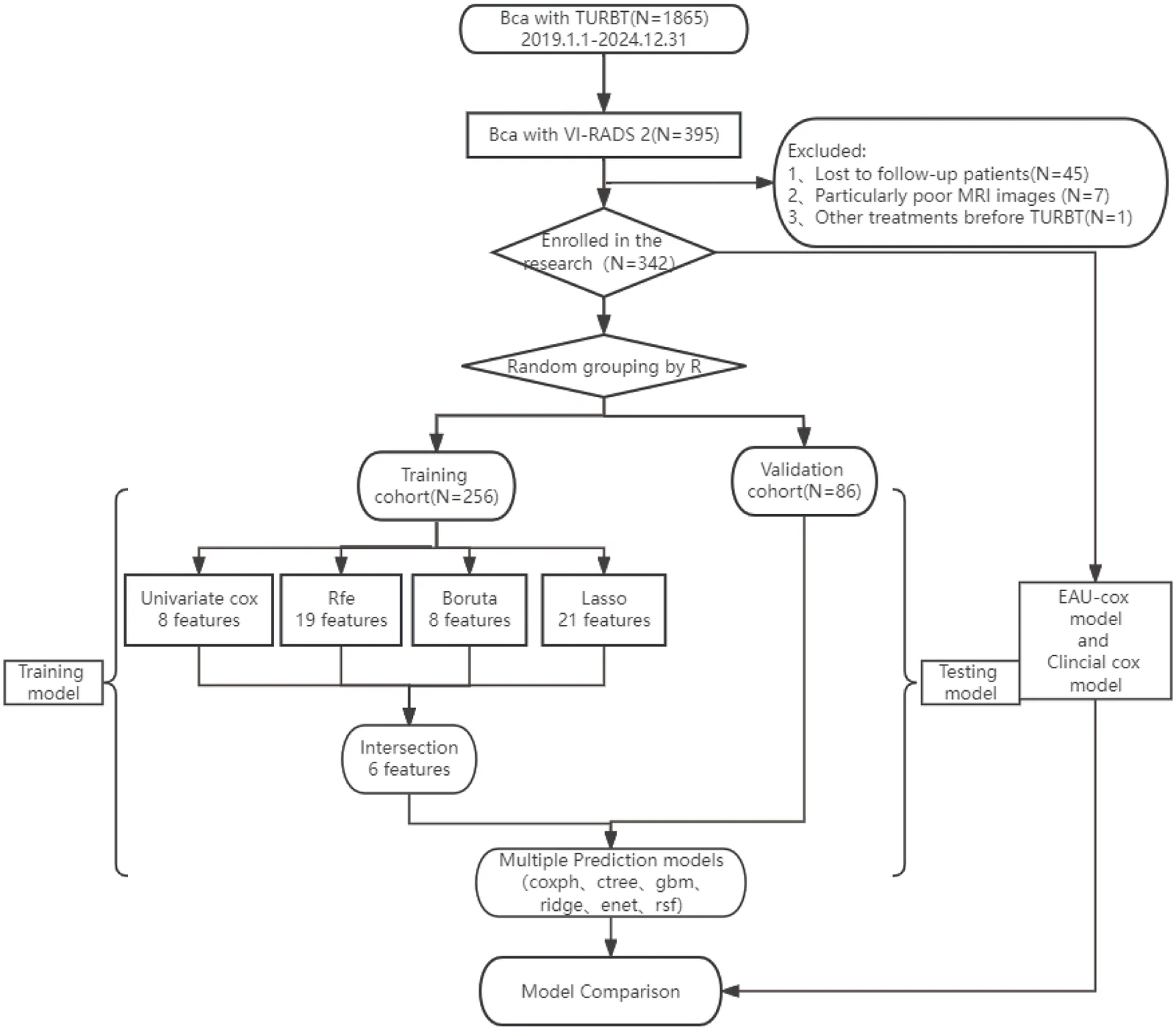
Technical roadmap.

A total of 342 eligible patients were randomly divided into a training cohort (n = 256) and a validation cohort (n = 86) at a ratio of 3:1. The training cohort was used for feature selection, model fitting, and hyperparameter tuning. The validation cohort was held out from these procedures and used for internal performance evaluation. The study workflow is shown in **Figure 1**.

### 2.2 MRI acquisition

MRI examinations were performed using a 3.0-T scanner (MAGNETOM Skyra; Siemens Healthineers, Erlangen, Germany). The protocol included axial T2-weighted imaging (T2WI) and DWI.

T2WI was acquired using the following parameters: repetition time, 4000 ms; echo time, 90 ms; slice thickness, 4 mm; interslice gap, 1 mm; field of view, 240 × 240 mm; and matrix size, 320 × 256.

DWI was acquired with b values of 0, 800, and 1000 s/mm². ADC maps were generated automatically on the workstation using a monoexponential decay model. All examinations followed a standardized bladder cancer MRI protocol.

### 2.3 Image analysis and feature extraction

#### 2.3.1 VI-RADS scoring

Two genitourinary radiologists with 8 and 15 years of experience independently assigned VI-RADS scores while blinded to clinical and pathological information. Disagreements were resolved by consensus. In patients with multiple tumors, the lesion with the greatest imaging burden, defined by the largest volume or greatest suspected depth of invasion, was selected as the target lesion.

#### 2.3.2 MRI morphologic features and ADC measurements

**Figure 2:**
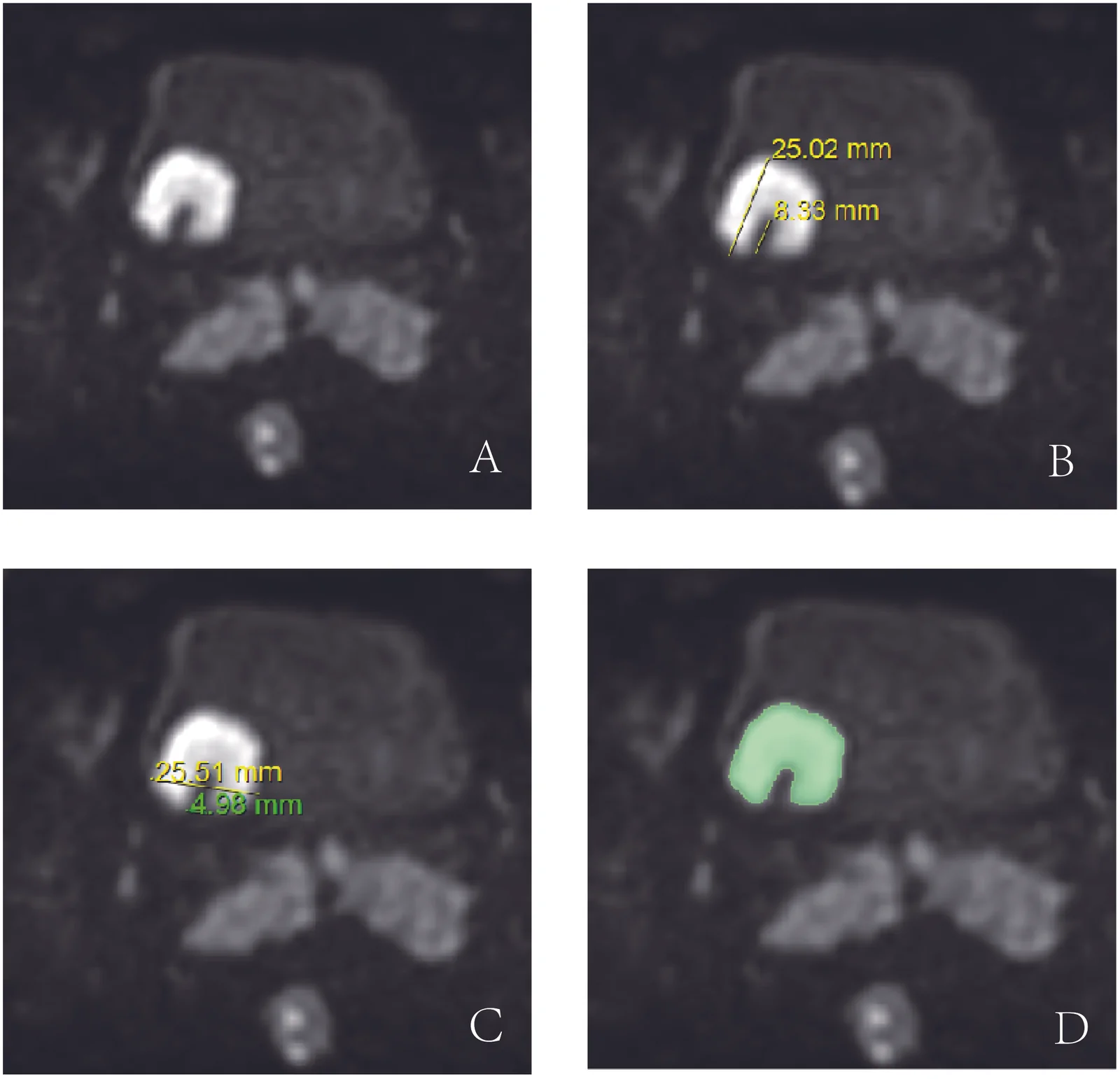
Typical DWI VI_RADS 2 cases and a diagram illustrating the measurement of quantitative parameters. **(A)** VI_RADS 2 tumor morphology: the tumor pedunculated and well_defined; postoperative pathology confirmed non_muscle_invasive bladder cancer (NMIBC); **(B)** The tumor's vertical length 25.02 mm (TVDmaxT) and the vertical length at the base is 8.33mm (TVDmaxB). **(C)** The tumor's maximum external diameter is 25.51 mm (TDmaxT), and the maximum distance between the base of the tumor stalk and the bladder wall is 4.98 mm (TCLmaxB). **(D)** Draw a region of interest (ROI) around the tumor portion of the bladder.

DWI served as the primary sequence for morphologic assessment. In patients with multiple tumors, measurements were obtained from the same target lesion used for VI-RADS scoring. The recorded morphologic variables were tumor-base morphology group (FBG; pedunculated, broad-based, or other), maximum tumor contact length (TCLmaxT), maximum tumor vertical distance (TVDmaxT), maximum tumor diameter (TDmaxT), tumor diameter group (TDmaxTG; ≤3 cm or >3 cm), pedicle/base vertical distance (TVDmaxB), and maximum contact length between the tumor base or pedicle and the bladder wall (TCLmaxB). For ADC assessment, the section that best depicted the tumor-bladder wall interface was selected. Regions of interest (ROIs) were manually placed in the exophytic tumor and the tumor base or pedicle, with necrotic and hemorrhagic areas excluded. Minimum, mean, maximum, and ADC minimum, mean, maximum, and within-ROI standard deviation were recorded for the tumor (ADCminT, ADCmeanT, ADCmaxT, and ADCmeanSDT) and the tumor base (ADCminB, ADCmeanB, ADCmaxB, and ADCmeanSDB). ADC values were reported to two decimal places in units of × 10^-3^ mm²/s. Two radiologists performed the measurements independently; each lesion was measured twice, and the mean of the repeated measurements was used for analysis. Measurements were obtained using Syngo.via (Siemens Healthineers), with the readers blinded to pathological outcomes and recurrence status. Interobserver agreement was assessed using intraclass correlation coefficients (ICCs). The measurement procedure is illustrated in **Figures 2A–D**.

### 2.4 Clinical variables

Clinical data were obtained from electronic health records. Candidate variables included age, age group (≤70 or >70 years), sex, smoking history, pathological stage (Ta or T1), tumor grade (low or high), tumor number (single or multiple), postoperative gemcitabine intravesical instillation, and repeat TURBT (ReTURBT). Intravesical instillation was coded as a binary variable according to whether regular postoperative gemcitabine instillation was administered.

Pathological T1 substaging (T1a/T1b/T1c) was not included as a candidate variable because it was not reported uniformly at our institution between 2019 and 2024. Complete T1 substage data were unavailable for more than 40% of the cohort, precluding reliable inclusion in multivariable analyses.

Patients were also classified as having low, intermediate, or high risk according to the EAU risk stratification system.

For comparison, an EAU-based Cox model was fitted with the EAU risk category as the sole predictor, and its performance was evaluated using the same metrics as the machine-learning models. Because the EAU system was developed in broader NMIBC populations rather than cohorts selected by VI-RADS score, this comparison was considered exploratory.

### 2.5 Feature selection

Feature selection combined statistical and machine-learning methods. Univariable Cox proportional hazards regression was first used to screen variables associated with RFS. Least absolute shrinkage and selection operator (LASSO)-penalized Cox regression, the Boruta algorithm, and recursive feature elimination (RFE) were then applied to reduce dimensionality. Variables selected by multiple methods were retained as candidate predictors for the final models.

All feature-selection procedures were performed exclusively in the training cohort (n = 256). The validation cohort was not used for feature selection, hyperparameter tuning, or model fitting.

The feature-selection results are shown in **Supplementary Figures 1A–E**.

### 2.6 Model development

Six survival prediction algorithms, including Cox proportional hazards regression, conditional inference tree (CTree), gradient boosting machine (GBM), ridge regression, elastic net regression, and random survival forest (RSF), were developed and compared. To minimize overfitting and prevent data leakage, all feature selection, model training, and hyperparameter optimization procedures were performed exclusively within the training cohort (n=256), while the validation cohort (n=86) was completely isolated and used only for independent model evaluation.

Feature selection was conducted using a sequential strategy combining univariate Cox regression, least absolute shrinkage and selection operator (LASSO), Boruta algorithm, and recursive feature elimination, reducing 22candidate variables to six stable predictors. Model hyperparameters were optimized using stratified 5-fold cross-validation and constrained grid search within the training set. Regularization strategies were applied according to algorithm characteristics, including coefficient shrinkage for penalized Cox models, learning rate and subsampling constraints for GBM, and node-size restriction for RSF to improve model generalizability.

Model performance was evaluated using Harrell’s concordance index (C-index), time-dependent area under the curve (AUC), Brier score, calibration curves, and decision curve analysis (DCA). Risk stratification thresholds were determined from the training cohort and directly applied to the validation cohort without recalculation. The degree of optimism was assessed by comparing model performance between training and validation cohorts. All analyses were performed using R software, with complete workflows for data preprocessing, feature selection, model construction, performance evaluation, and model interpretation documented to ensure reproducibility.

### 2.7 Internal validation and reproducibility

Model performance was evaluated separately in the training and held-out validation cohorts. Risk-group cutoffs were derived exclusively from the training cohort and applied unchanged to the validation cohort. The difference between training and validation performance was used descriptively to assess optimism. Complete R code for data processing, feature selection, model fitting, hyperparameter tuning, Shapley additive explanations (SHAP), and performance evaluation is available from the corresponding author upon reasonable request; the required R packages are documented in the **Supplementary Material**.

### 2.8 Model evaluation

Discrimination was assessed using the C-index and time-dependent receiver operating characteristic curves, with AUCs calculated at 12, 24, 36, and 60 months. Calibration plots were used to compare predicted and observed RFS probabilities, and Brier scores were calculated to assess overall prediction error. Decision curve analysis (DCA) was performed at 60 months to estimate net benefit across a range of threshold probabilities.

### 2.9 Statistical analysis

Statistical analyses were performed using R version 4.5.1. Normally distributed continuous variables are presented as the mean ± standard deviation and were compared using an independent-samples t test. Skewed continuous variables are presented as the median (first quartile, third quartile) and were compared using a rank-sum test. Categorical variables are presented as n (%) and were compared using the chi-square test or Fisher’s exact test, as appropriate. All tests were two-sided, and P < 0.05 was considered statistically significant. Time-dependent AUC and calibration analyses used inverse probability of censoring weighting (IPCW) to account for right censoring.

Interobserver agreement for quantitative imaging measurements was assessed using ICCs estimated from a two-way mixed-effects model. ICCs were interpreted as excellent (≥0.80), good (0.61-0.79), fair (0.41-0.60), poor (0.21-0.40), or very poor (≤0.20).

Missing clinical and imaging variables were assessed before analysis. Patients with incomplete essential information were excluded according to the prespecified criteria; therefore, no imputation was performed.

## 3 Results

### 3.1 Patient characteristics

**Figure 3:**
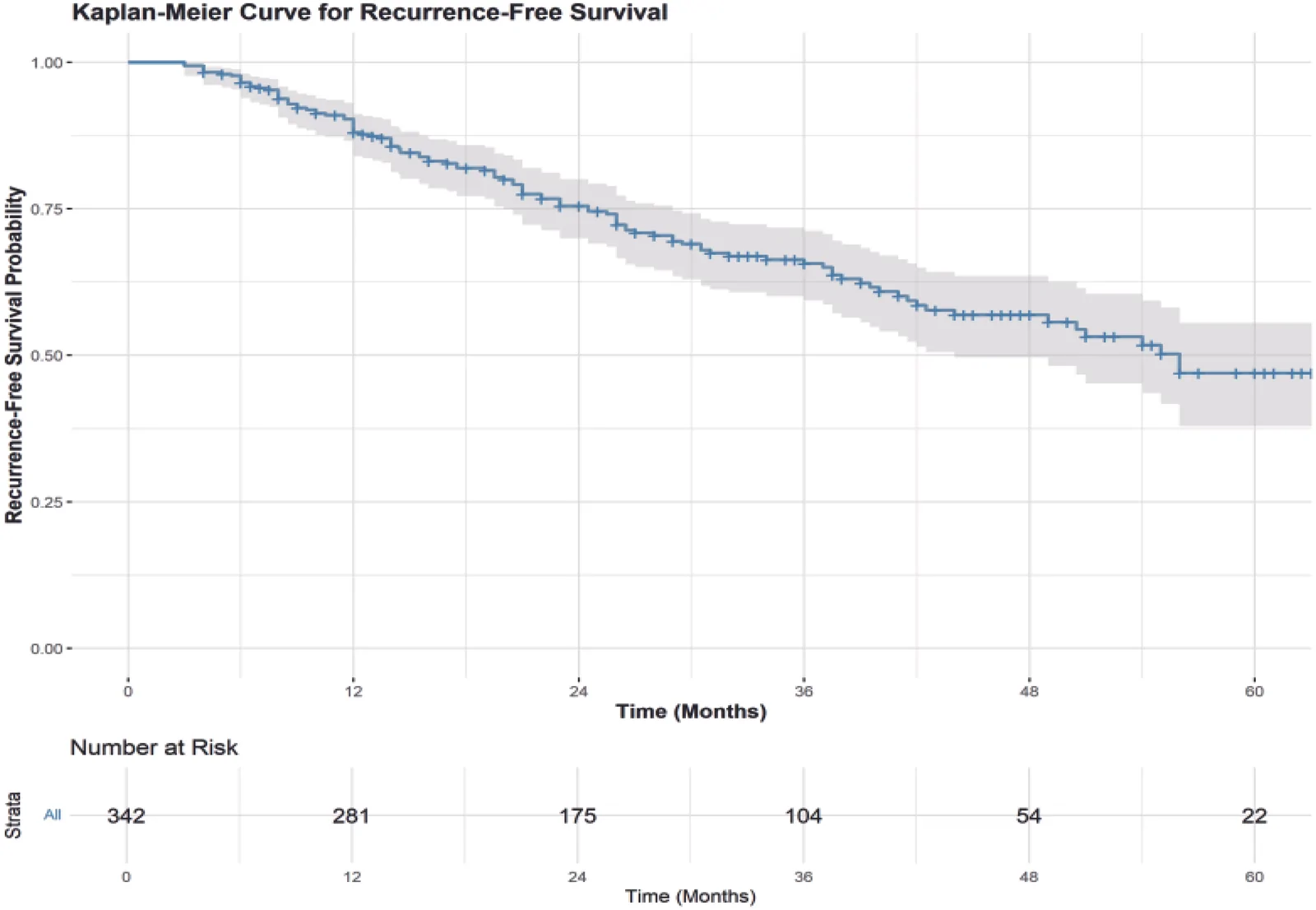
Kaplan–Meier (KM) curves for all patients.

Of the 342 patients, 72 had a follow-up duration of at least 60 months. The numbers remaining at risk at 12, 24, 36, and 60 months were 281, 175, 104, and 22, respectively. The estimated 5-year censoring proportion was 78.9%. Detailed numbers at risk and censoring counts are provided in **Supplementary Table 2**, and the Kaplan–Meier curve for the overall cohort is shown in **Figure 3**.

Baseline clinical and imaging characteristics were similar between the training and validation cohorts, with no statistically significant differences in age, sex, pathological stage, tumor grade, tumor size, tumor number, or EAU risk category (all P > 0.05; **Table 1**).

**Table 1:** Baseline characteristics of VI-RADS 2 NMIBC patients: training vs. validation sets.

Variable	Training set (n = 256)¹	Validation set (n = 86)¹	P value²
age	63.7 (12.3)	63.9 (12.5)	0.9
AG			0.6
≤70 years	182 (71.1%)	58 (67.4%)	
>70 years	74 (28.9%)	28 (32.6%)	
grading			0.6
Low grade	99 (38.7%)	37 (43.0%)	
High grade	157 (61.3%)	49 (57.0%)	
staging			0.4
Ta	113 (44.1%)	43 (50.0%)	
T1	143 (55.9%)	43 (50.0%)	
NTG			0.4
Single	197 (77.0%)	62 (72.1%)	
Multiple	59 (23.0%)	24 (27.9%)	
II			0.9
No	67 (26.2%)	24 (27.9%)	
Yes	189 (73.8%)	62 (72.1%)	
ReTURBT			>0.9
No	193 (75.4%)	66 (76.7%)	
Yes	63 (24.6%)	20 (23.3%)	
RG			0.060
Low risk	79 (30.9%)	35 (40.7%)	
Intermediate risk	61 (23.8%)	11 (12.8%)	
High risk	116 (45.3%)	40 (46.5%)	
TDmaxTG			0.5
≤3cm	207 (80.9%)	73 (84.9%)	
>3cm	49 (19.1%)	13 (15.1%)	
FBG			0.3
Pedunculated	67 (26.2%)	16 (18.6%)	
Broad-based	95 (37.1%)	31 (36.0%)	
Other	94 (36.7%)	39 (45.3%)	
TVDmaxT	18.3 (7.9)	16.9 (7.1)	0.11
TDmaxT	23.4 (9.4)	22.0 (7.9)	0.2
TVDmaxB	8.7 (4.1)	8.1 (4.0)	0.3
TCLmaxB	8.4 (5.9)	7.7 (5.1)	0.4
ADCminB	1.5 (0.3)	1.5 (0.3)	>0.9
ADCmaxB	2.2 (0.5)	2.1 (0.4)	0.18
ADCmeanSDB	0.23 (0.11)	0.21 (0.10)	0.15
ADCmeanB	1.9 (0.4)	1.9 (0.3)	0.2
ADCminT	1.0 (0.3)	0.9 (0.3)	0.3
ADCmaxT	2.3 (0.4)	2.2 (0.3)	0.08
ADCmeanSDT	0.27 (0.12)	0.25 (0.11)	0.13
ADCmeanT	1.5 (0.3)	1.4 (0.3)	0.062

¹Continuous variables are presented as mean (SD); categorical variables as n (%).

²Welch Two Sample t-test; Pearson’s Chi-squared test.

AG, Age group (≤70 years, >70 years).

grading, Pathological grade (Low grade, High grade).

staging, T stage (Ta, T1).

NTG, Tumor number (Single, Multiple).

II, Intravesical instillation (No, Yes).

ReTURBT, Second transurethral resection (No, Yes).

RG, EAU risk group (Low, Intermediate, High).

TDmaxTG, Tumor diameter group (≤3cm, >3cm).

FBG, Base morphology (Pedunculated, Broad-based, Other).

TVDmaxT, Tumor vertical distance (cm).

TDmaxT, Tumor maximum diameter (cm).

TVDmaxB, Pedicle vertical distance (cm).

TCLmaxB, Base contact length (cm).

ADCminB/ADCmeanB/ADCmaxB/ADCmeanSDB, Minimum/mean/maximum/SD of ADC at base (×10⁻³ mm²/s).

ADCminT/ADCmeanT/ADCmaxT/ADCmeanSDT, Minimum/mean/maximum/SD of ADC at tumor (×10⁻³ mm²/s).

NMIBC, Non-muscle invasive bladder cancer.

ADC, Apparent diffusion coefficient.

TURBT, Transurethral resection of bladder tumor.

EAU, European Association of Urology.

ICCs for the quantitative imaging variables ranged from 0.826 to 0.988, indicating excellent interobserver agreement. The ICCs were 0.966 for TVDmaxB, 0.977 for TCLmaxB, 0.986 for ADCminB, and 0.988 for TDmaxT.

### 3.2 Follow-up and recurrence outcomes

During a median follow-up of 24.75 months, 111 patients (32.5%) experienced recurrence. The RFS curve for the overall cohort is shown in **Figure 3**.

### 3.3 Feature selection

Twenty-two clinical and quantitative MRI variables were initially considered. Univariable Cox analysis identified potential associations with RFS (P < 0.05) for several variables, including tumor number, tumor size, and ADC-related measurements. LASSO, Boruta, and RFE further reduced the feature set. Six variables were retained across the selection procedures: tumor number, maximum tumor vertical distance (TVDmaxT), maximum tumor diameter (TDmaxT), maximum tumor-base contact length (TCLmaxB), mean ADC at the tumor base (ADCmeanB), and minimum ADC within the tumor (ADCminT).

### 3.4 Model performance in the training cohort

In the training cohort, GBM achieved the highest C-index (0.836), followed closely by RSF (0.832). The C-index values were 0.782 for ridge regression, 0.781 for the Cox model, 0.779 for elastic net regression, and 0.755 for CTree. RSF also showed consistently favorable time-dependent AUCs and was selected for detailed evaluation because it maintained a better balance between training and validation performance than GBM.

Time-dependent AUCs for all models in the training cohort are shown in **Supplementary Figures 2A–F**.

### 3.5 Model performance in the validation cohort

C-index values with 95% confidence intervals for all models in the training and validation cohorts are summarized in **Table 2**.

**Table 2:** C-index performance of survival prediction models in the training and validation sets.

Model_Abbreviation	Training_Cindex	Training_Cindex_CI	Validation_Cindex	Validation_Cindex_CI
coxph	0.7823685	0.7400363 - 0.8243716	0.7218586	0.6221347 - 0.8211864
ctree	0.7549043	0.7094977 - 0.8080334	0.6999345	0.5933111 - 0.7989113
enet	0.7785251	0.7326194 - 0.8255756	0.7205498	0.6177490 - 0.8148546
gbm	0.8362159	0.7998762 - 0.8735231	0.7627618	0.6721507 - 0.8445507
ridge	0.7819681	0.7397980 - 0.8258229	0.7277487	0.6323322 - 0.8238874
rsf	0.8323324	0.7972058 - 0.8720300	0.7571989	0.6758918 - 0.8301420

C-index, concordance index; CI, 95% confidence interval.

coxph, Cox proportional hazards model; ctree, conditional inference tree; enet, elastic net Cox model; gbm, gradient boosting model; ridge, ridge Cox model; rsf, random survival forest.

In the validation cohort, the RSF model achieved a C-index of 0.757. Its time-dependent AUCs were 0.761, 0.818, 0.775, and 0.789 at 12, 24, 36, and 60 months, respectively (**Supplementary Figures 3A–F**). The GBM model showed a larger decline from training to validation performance, suggesting overfitting, whereas the Cox, ridge, elastic net, and CTree models had lower validation performance. Time-dependent AUCs, 95% confidence intervals, and Brier scores are presented in **Table 3**. Because the numbers at risk in the overall cohort declined to 104 at 36 months and 22 at 60 months, the long-term estimates, particularly those at 60 months, should be interpreted cautiously. External validation is required to assess generalizability.

**Table 3:** Performance comparison of different survival prediction models at 12-, 24-, 36-, and 60-month time points.

Model_Abbreviation	Time_point	Training_AUC	Training_AUC_CI	Test_AUC	Test_AUC_CI	Training_Brier	Validation_Brier
coxph	12	0.831	0.729-0.932	0.81	0.679-0.940	0.085	0.115
coxph	24	0.809	0.722-0.896	0.767	0.606-0.928	0.14	0.185
coxph	36	0.778	0.683-0.873	0.753	0.577-0.928	0.175	0.19
coxph	60	0.906	0.791-1.020	0.815	0.639-0.990	0.13	0.19
ctree	12	0.848	0.769-0.927	0.733	0.503-0.963	0.085	0.115
ctree	24	0.759	0.663-0.854	0.731	0.577-0.884	0.14	0.185
ctree	36	0.756	0.658-0.853	0.695	0.522-0.869	0.175	0.19
ctree	60	0.899	0.817-0.981	0.744	0.531-0.957	0.13	0.19
enet	12	0.819	0.713-0.925	0.793	0.645-0.940	0.085	0.115
enet	24	0.805	0.717-0.894	0.771	0.613-0.928	0.14	0.185
enet	36	0.775	0.679-0.870	0.738	0.557-0.919	0.18	0.19
enet	60	0.903	0.787-1.020	0.82	0.644-0.995	0.12	0.19
gbm	12	0.891	0.819-0.963	0.799	0.638-0.961	0.12	0.115
gbm	24	0.865	0.793-0.936	0.821	0.687-0.955	0.17	0.22
gbm	36	0.845	0.761-0.929	0.782	0.609-0.956	0.22	0.23
gbm	60	0.947	0.870-1.024	0.844	0.676-1.013	0.25	0.27
ridge	12	0.824	0.720-0.928	0.81	0.680-0.941	0.085	0.115
ridge	24	0.807	0.720-0.895	0.78	0.625-0.935	0.14	0.185
ridge	36	0.777	0.682-0.871	0.751	0.575-0.927	0.175	0.19
ridge	60	0.908	0.794-1.023	0.825	0.657-0.993	0.125	0.19
rsf	12	0.883	0.808-0.957	0.761	0.589-0.933	0.08	0.115
rsf	24	0.864	0.796-0.932	0.818	0.690-0.945	0.12	0.175
rsf	36	0.846	0.763-0.929	0.775	0.604-0.946	0.13	0.185
rsf	60	0.939	0.860-1.018	0.789	0.562-1.015	0.12	0.25

AUC, area under the receiver operating characteristic curve; CI, confidence interval; Brier score, prediction error (lower values indicate better calibration).

coxph, Cox proportional hazards model; ctree, conditional inference tree; enet, elastic net Cox model; gbm, gradient boosting model; ridge, ridge Cox model; rsf, random survival forest.

Time-dependent AUC and Brier scores were calculated via inverse probability of censoring weighting (IPCW) to handle incomplete long-term follow-up.

### 3.6 Model calibration and overall performance

Calibration plots showed approximate agreement between predicted and observed RFS probabilities for RSF in both cohorts. Agreement was less certain at later time points because fewer patients remained at risk.

**Figure 4:**
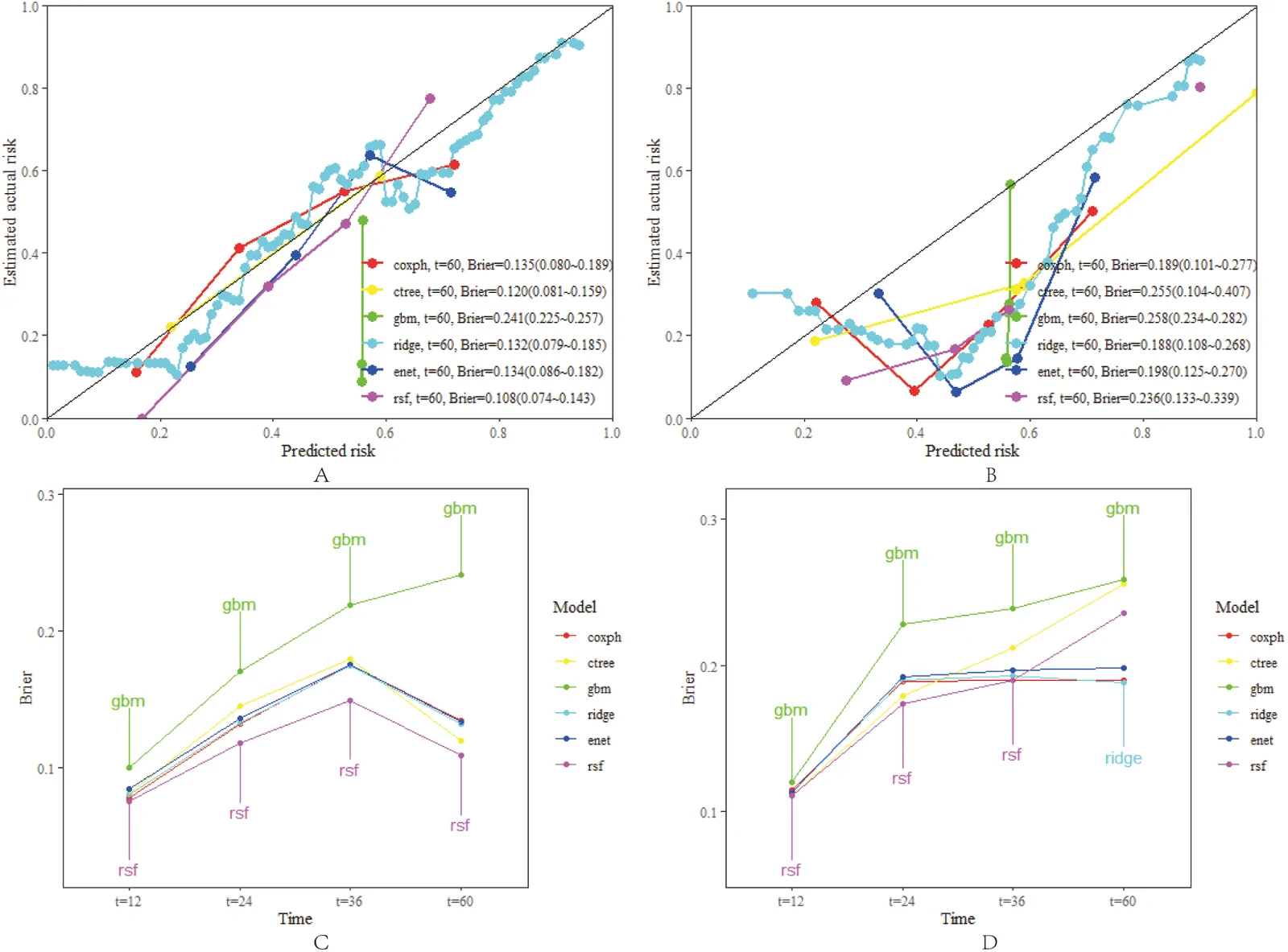
**(A)** Calibration plots of different prediction models at t=60 in the training set. **(B)** Calibration plots of different prediction models at t=60in the test set. **(C)** Brier score curves of different models at different time points in the training set. **(D)** Brier score curves of different models at different time points in the test set.

RSF also had lower Brier scores than the other models across the evaluated time points in both cohorts, indicating lower overall prediction error (**Figures 4A–D**).

### 3.7 Comparison with clinical and guideline-based risk models

A clinical Cox model based on routinely available clinicopathological variables was constructed as a reference model. In the validation cohort, the C-index was 0.757 for the MRI-based RSF model, 0.417 for the clinical Cox model, and 0.534 for the EAU-based model.

**Figure 5:**
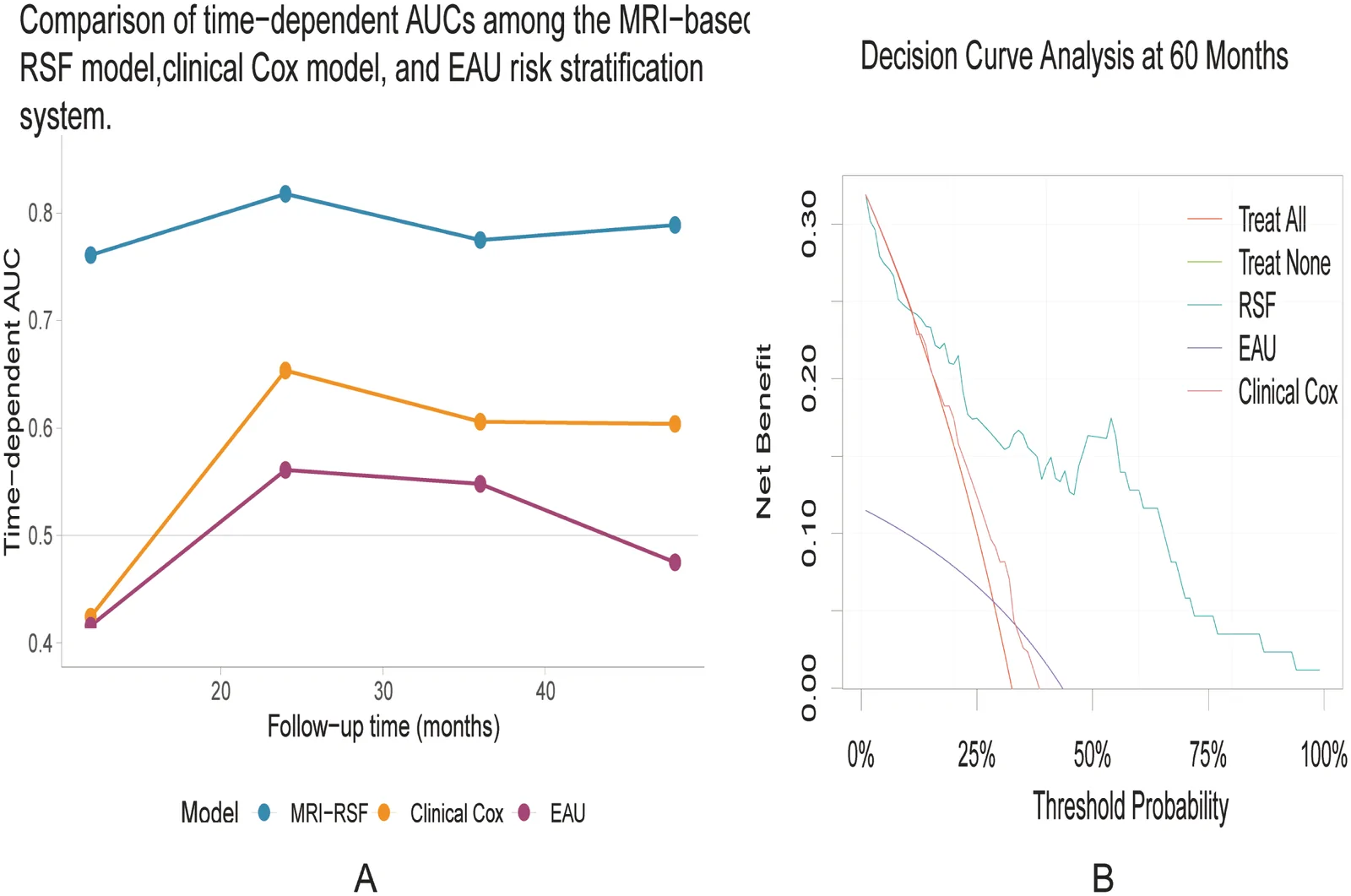
**(A)** Time-dependent AUC (RSF vs EAU vs Clinical Cox model). Time-dependent AUC and calibration estimates were adjusted by inverse probability of censoring weighting to accommodate right censoring and partial follow-up data. **(B)** DCA (RSF vs EAU vs Clinical Cox model 60month).

The MRI-based RSF model also yielded higher time-dependent AUCs than both reference models at all evaluated time points (**Figure 5A**). At 12, 24, 36, and 60 months, the AUCs were 0.761, 0.818, 0.775, and 0.789 for RSF; 0.423, 0.641, 0.590, and 0.598 for the clinical Cox model; and 0.353, 0.458, 0.434, and 0.418 for the EAU-based model, respectively. At 60 months, DCA indicated greater net benefit for RSF across a range of threshold probabilities (**Figure 5B**), although this analysis was based on a limited number of patients remaining at risk. DCA curves for the training and validation cohorts are shown in **Supplementary Figures 4A, B**.

These findings suggest that quantitative MRI features may capture recurrence-related heterogeneity within the VI-RADS 2 subgroup that is not fully represented by conventional clinicopathological variables or guideline-based categories.

### 3.8 Model interpretability

Variable-importance analysis identified ADCminT, ADCmeanB, and tumor number as the most influential predictors in the RSF model, with smaller contributions from the remaining imaging variables.

**Figure 6:**
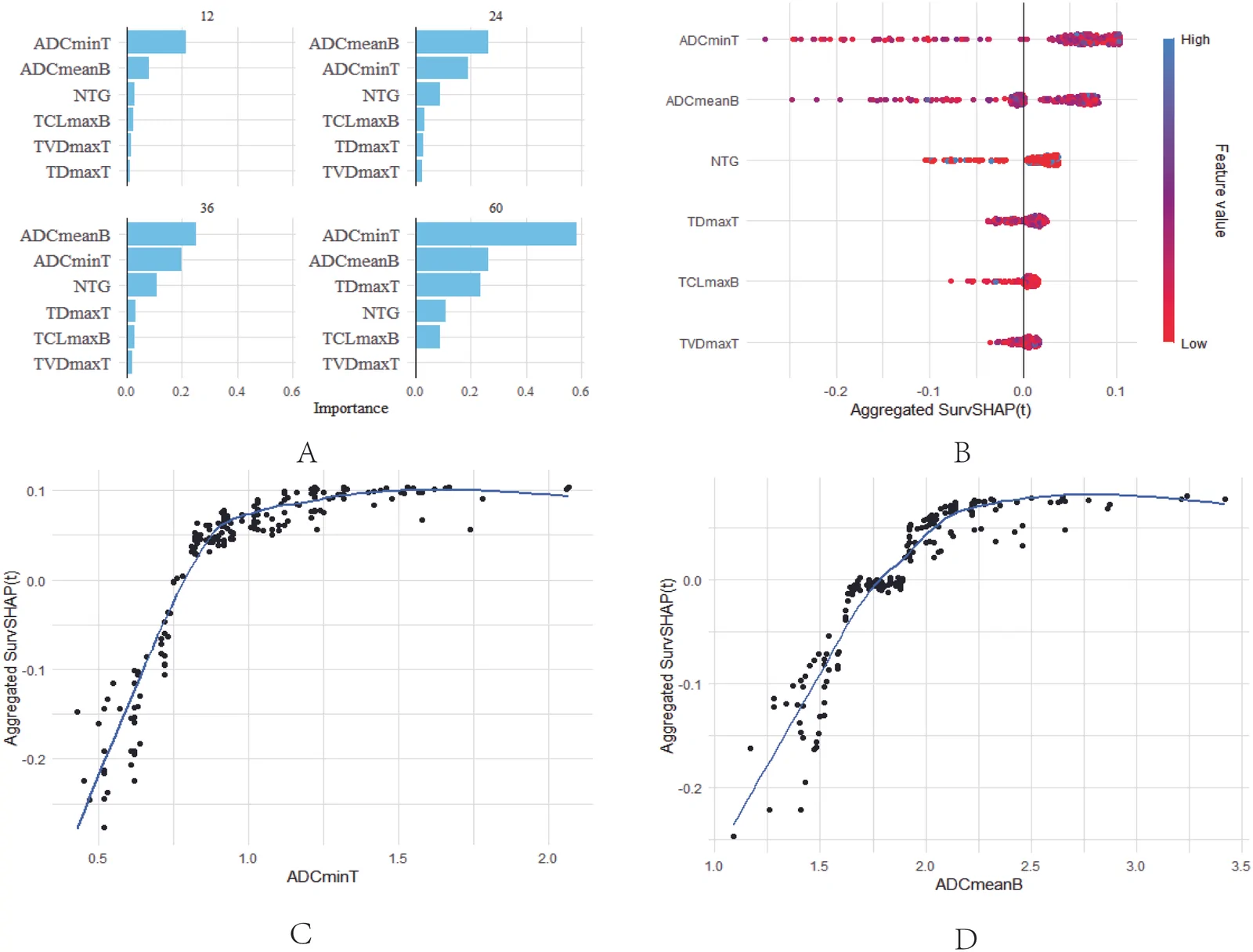
**(A)** Variable importance ranking of predictors in the RSF model at different time points. **(B)** SHAP summary plot illustrating the contribution and direction of each variable to recurrence risk. **(C, D)** Partial dependence plots showing the relationship between ADC-derived features (ADCminT and ADCmeanB) and predicted recurrence risk.

SHAP analysis indicated that lower ADC values were associated with higher model-predicted recurrence risk. Partial dependence plots suggested nonlinear relationships between ADC-derived variables and predicted recurrence risk (**Figures 6A–D**). These model-based associations should not be interpreted as causal effects.

### 3.9 RSF-based risk stratification

**Figure 7:**
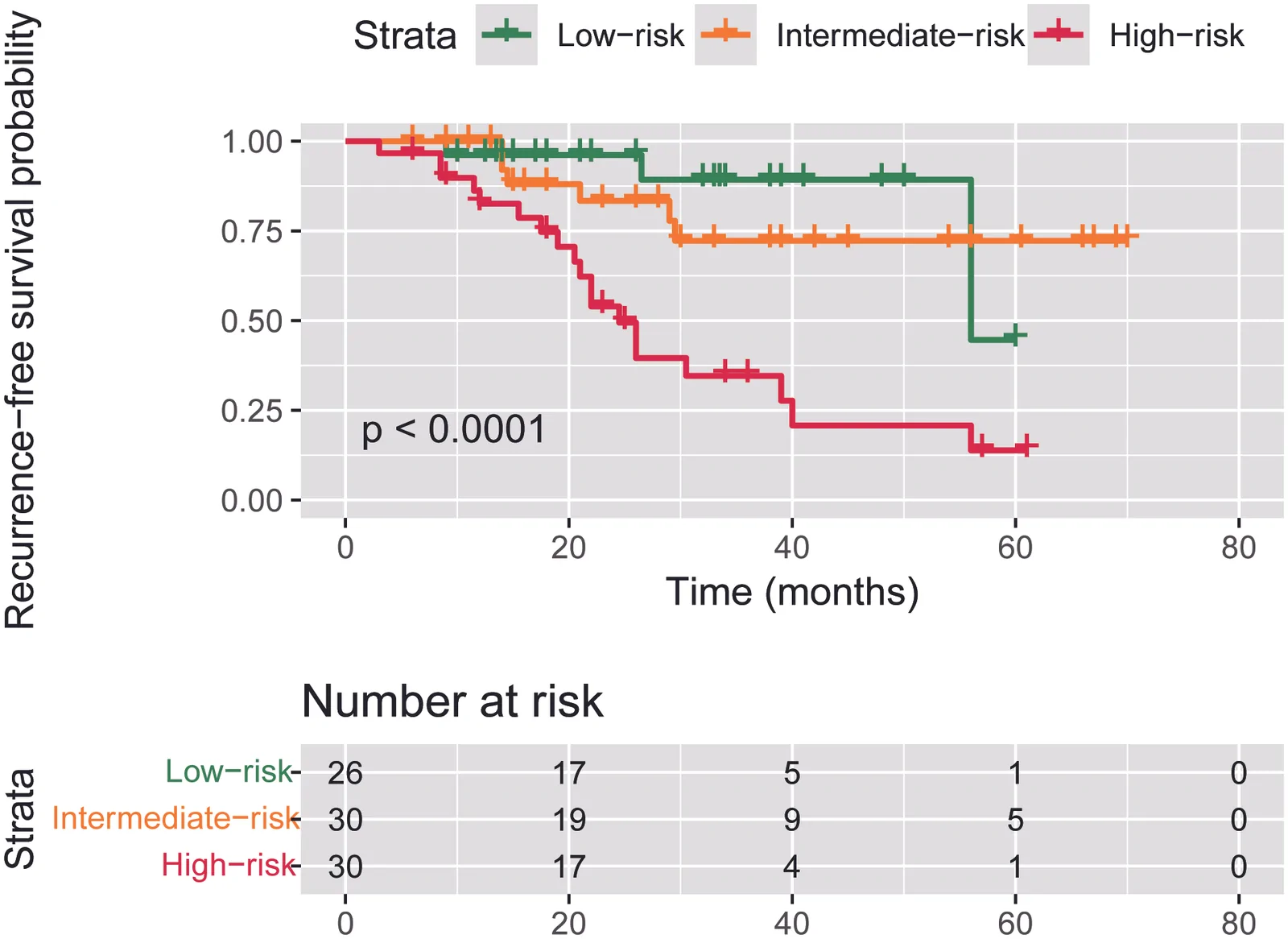
Kaplan–Meier curves of recurrence-free survival according to RSF-derived risk groups. Patients were classified into low-, intermediate-, and high-risk groups according to RSF-derived recurrence risk scores. The cutoff values were determined in the training cohort and applied to the validation cohort. Differences among groups were assessed using log-rank tests.

Patients were stratified into low-, intermediate-, and high-risk groups using RSF-derived risk scores. The cutoffs (7.2361 and 22.8953) were derived in the training cohort and applied unchanged to the validation cohort. RFS differed among the three groups in both cohorts (log-rank P < 0.001 in the training cohort and P = 2.695 × 10^-5^ in the validation cohort). Compared with the low-risk group, the high-risk group had a higher recurrence hazard in the training cohort (hazard ratio [HR], 7.32; 95% confidence interval [CI], 4.62-11.58) and the validation cohort (HR, 5.30; 95% CI, 2.39-11.76; **Figure 7**). These findings demonstrate risk-group separation within the study cohort.

## 4 Discussion

In this single-center retrospective study, recurrence risk varied substantially among patients with VI-RADS 2 NMIBC. A model incorporating tumor number and quantitative MRI features showed higher internal discrimination than a clinical Cox model and an EAU-based model. ADC-derived and morphologic variables contributed to risk prediction and showed nonlinear associations with model output. These findings support further evaluation of MRI-derived measurements as potential adjuncts to conventional risk assessment.

Previous studies have evaluated mpMRI, VI-RADS, and radiomics for recurrence prediction or local staging in broader bladder cancer populations (8, 14, 15). Xu et al. reported that higher VI-RADS scores were associated with recurrence risk, but categorical scoring does not address heterogeneity within the VI-RADS 2 subgroup. Lower pretreatment ADC values have also been associated with recurrence after TURBT (16). The present study extends this work by evaluating manually measurable tumor morphology and ADC variables specifically in patients with VI-RADS 2 NMIBC and by comparing several survival-modeling approaches. The use of a limited set of quantitative variables may be more readily reproducible than high-dimensional radiomic signatures, although this requires confirmation across scanners and institutions.

VI-RADS 2 indicates a low likelihood of muscularis propria invasion, but it was not designed as a recurrence-risk classification system (17, 18). The separation of RFS curves in the present cohort suggests that recurrence outcomes may remain heterogeneous even within this imaging category. This observation should not be interpreted as changing the intended staging role of VI-RADS or as replacing clinicopathological risk assessment.

Among the evaluated algorithms, RSF provided the most favorable balance between training and validation performance. Its validation C-index of 0.757 and favorable time-dependent AUCs indicate useful discrimination within this cohort; however, the decrease from the training C-index of 0.832 suggests some optimism. RSF can model nonlinear effects and interactions without prespecifying their functional form, which may be advantageous for quantitative imaging variables. Nevertheless, its performance cannot be assumed to generalize beyond the present institution.

The clinical Cox model showed limited discrimination in the validation cohort. One possible explanation is the restricted study population: all patients had VI-RADS 2 NMIBC, which may have reduced variability in conventional stage- and grade-related predictors. ADC-derived measurements may provide complementary information on cellularity and extracellular space (10, 12). However, the observed performance difference reflects both predictor composition and modeling method and therefore does not isolate the independent contribution of MRI features.

The RSF model yielded higher C-index and time-dependent AUC values than the clinical Cox and EAU-based models in the validation cohort. DCA also suggested a higher net benefit at 60 months. These comparisons indicate potential incremental value within the study cohort but should be viewed as exploratory, particularly because the 60-month analysis was based on limited long-term follow-up.

The lower performance of the reference models may also reflect their coarser representation of risk in an imaging-restricted subgroup. Quantitative MRI variables are continuous and may capture morphologic and microstructural variation that is not represented by broad clinical categories. In addition, RSF can accommodate nonlinear effects and interactions. These factors may have contributed to its higher internal performance.

The comparisons require caution. The RSF and clinical Cox models differed in both predictors and statistical form, and the EAU system was developed for broad NMIBC populations rather than specifically for patients with VI-RADS 2 lesions (19). The present findings therefore support evaluation of MRI-derived variables as complementary biomarkers, not replacement of established clinical or guideline-based systems.

Calibration plots and Brier scores suggested acceptable agreement and overall prediction accuracy within the training and validation cohorts. However, calibration was assessed only internally, and uncertainty increased at later time points. The predicted absolute risks should therefore not be considered clinically transportable until external calibration and validation have been completed.

ADCminT and ADCmeanB were among the most influential RSF predictors. Lower ADC values were associated with higher predicted recurrence risk, which is biologically plausible because restricted water diffusion may reflect increased cellularity and reduced extracellular space (5, 6, 12). Partial dependence plots suggested nonlinear effects, but the apparent thresholds are exploratory and may be unstable in other datasets (20). SHAP and partial dependence analyses describe model behavior and do not establish causal relationships. Current imaging-based assessment generally considers VI-RADS 2 lesions as having a low likelihood of muscle invasion, and many patients subsequently undergo relatively similar management pathways based on conventional clinical risk assessment. However, our findings demonstrate substantial recurrence heterogeneity within this subgroup, with an overall recurrence rate of 32.5%. The proposed RSF model provides additional quantitative risk stratification by integrating MRI-derived biomarkers, particularly ADCminT, TCLmaxB, and tumor number.Clinically, this approach may help identify VI-RADS 2 patients with unfavorable biological characteristics despite apparently low-risk imaging classification. Patients with higher predicted recurrence risk may potentially benefit from closer surveillance and individualized postoperative management, whereas patients with favorable MRI features may avoid unnecessary intensive follow-up. However, these potential applications should be considered hypothesis-generating, and prospective studies are required to determine whether MRI-based risk stratification can safely guide surveillance strategies or intravesical treatment decisions. Therefore, the major clinical contribution of this model is not to replace current guideline-based management but to refine risk assessment within a population currently considered relatively homogeneous. By revealing hidden recurrence heterogeneity among VI-RADS 2 lesions, MRI-based modeling may provide a foundation for future personalized management strategies.

Several limitations should be acknowledged. First, this was a single-center retrospective study with internal validation based on random split-sampling rather than temporal or external multicenter validation. Although the validation cohort was completely independent during model development, variations in MRI acquisition protocols, patient characteristics, and clinical practices across institutions may affect model generalizability. Future prospective multicenter studies are required to confirm the robustness and clinical applicability of the proposed model. Second, the relatively limited sample size may have restricted the generalizability of our findings. Although multiple strategies were applied to reduce overfitting, including feature selection and cross-validation, larger cohorts with longer follow-up are needed for further validation, particularly for long-term recurrence prediction. Third, T1 substaging, an emerging pathological prognostic factor in NMIBC, was unavailable in our dataset because standardized T1 substage assessment was not routinely performed throughout the study period. Future studies incorporating comprehensive pathological parameters together with MRI-derived biomarkers may further improve risk stratification performance (21). Fourth, this study focused on conventional quantitative MRI measurements and did not incorporate high-dimensional radiomics or deep learning-based features. Additionally, tumor regions of interest were manually delineated, which may introduce observer-dependent variability. Automated segmentation and advanced artificial intelligence approaches should be explored in future investigations to improve reproducibility (17).Fifth, although intravesical therapy was considered, detailed treatment-related information, including induction schedules, maintenance regimens, and cumulative chemotherapy exposure, was unavailable. Therefore, potential residual confounding caused by treatment heterogeneity cannot be completely excluded. Finally, the median follow-up duration was relatively short (24.75 months), and the number of patients with long-term follow-up was limited. Although censoring-adjusted methods were applied, the accuracy of 60-month predictive estimates may be affected by limited event information. Longer follow-up is warranted to validate the model’s performance for late recurrence prediction.

**Figure 8:**
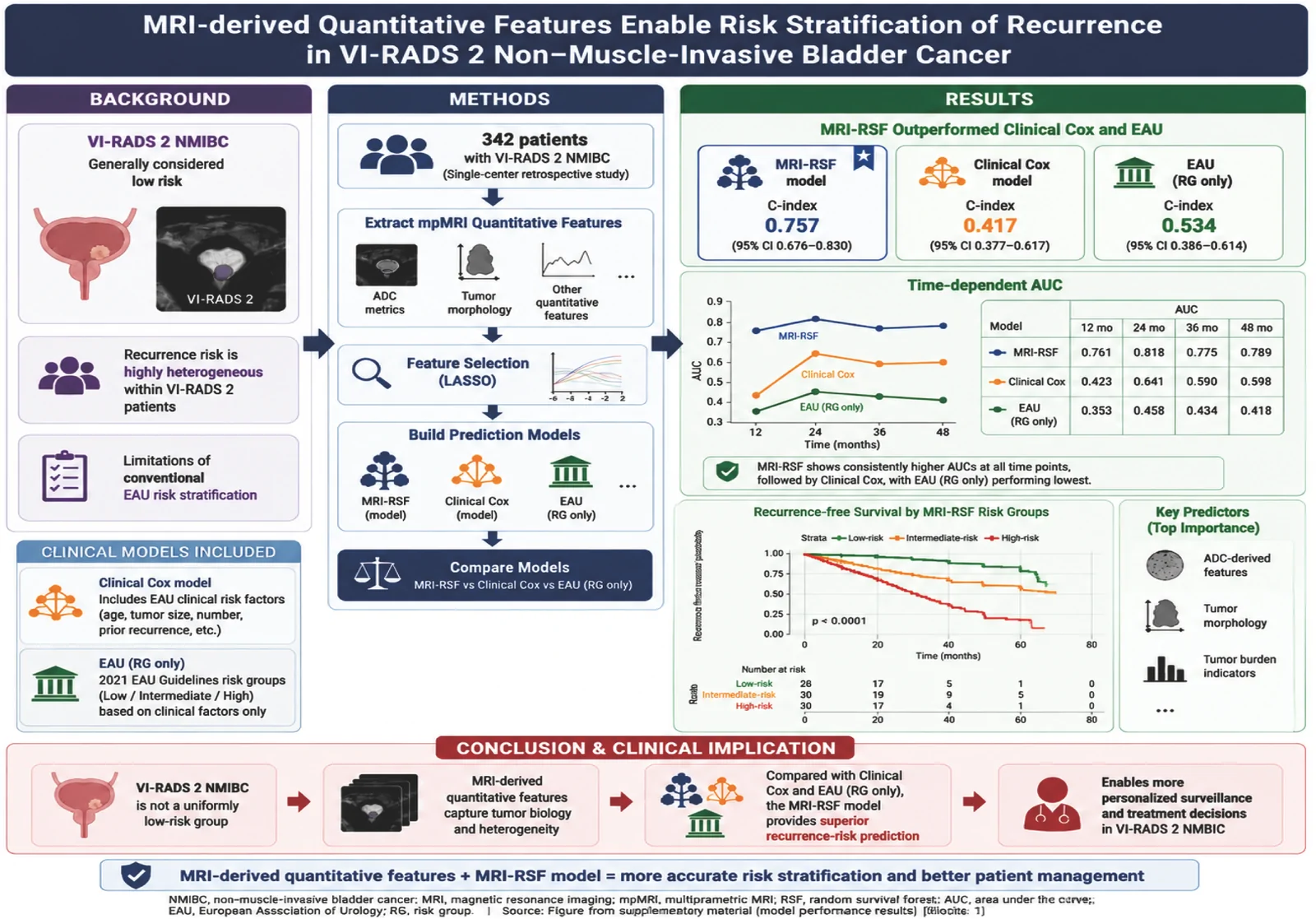
Graphical summary of the study design and main findings. Quantitative multiparametric MRI features were integrated with survival machine learning to stratify recurrence risk in 342 patients with VI‑RADS 2 NMIBC. The RSF model showed superior internal predictive performance compared with clinical and EAU‑based risk stratification and identified substantial recurrence heterogeneity within the VI‑RADS 2 subgroup. These findings support the potential of quantitative MRI biomarkers as complementary tools for refined recurrence risk stratification, although prospective multicenter external validation is required before clinical implementation.

**Figure 8** provides a graphical summary of the study design, analytical workflow, and main findings.

## 5 Conclusion

Patients with VI-RADS 2 NMIBC showed substantial heterogeneity in recurrence risk within this single-center cohort. An RSF model incorporating tumor number and quantitative MRI-derived variables achieved improved internal discrimination compared with clinical Cox and EAU-based models, suggesting that MRI-derived biomarkers may provide complementary prognostic information beyond conventional risk assessment. The RSF model further stratified patients into low-, intermediate-, and high-risk groups with distinct recurrence-free survival patterns, indicating its potential value in identifying hidden recurrence heterogeneity within the VI-RADS 2 subgroup. However, these findings are hypothesis-generating and should not be interpreted as supporting replacement of established risk systems or immediate changes in surveillance or treatment strategies. The retrospective single-center design, random split-sample internal validation, limited long-term follow-up, and absence of external validation restrict generalizability. Prospective multicenter studies with standardized MRI protocols, comprehensive clinical variables, and longer follow-up are required to validate model robustness and determine whether MRI-informed risk stratification can provide meaningful clinical benefit.

## Data Availability

The datasets generated and analyzed during the present study are not publicly available due to hospital patient privacy protection regulations. Anonymized analytical datasets can be obtained from the corresponding author upon reasonable written request after ethical approval. Requests to access the datasets should be directed to Hong Yang, yanghong0216@126.com.
