# Supplementary material for: MRI-derived features reveal recurrence heterogeneity in VI-RADS 2 non-muscle-invasive bladder cancer
Source: Front Oncol. 2026 Sep 3;16:1921569. doi: 10.3389/fonc.2026.1921569 (PMC13581660; doi:10.3389/fonc.2026.1921569)
Supplement: Supplementary file 1 [file DataSheet1.pdf]

Supplementary Information

Title: MRI-derived features reveal recurrence heterogeneity in VI-RADS 2 non-muscle-invasive bladder cancer

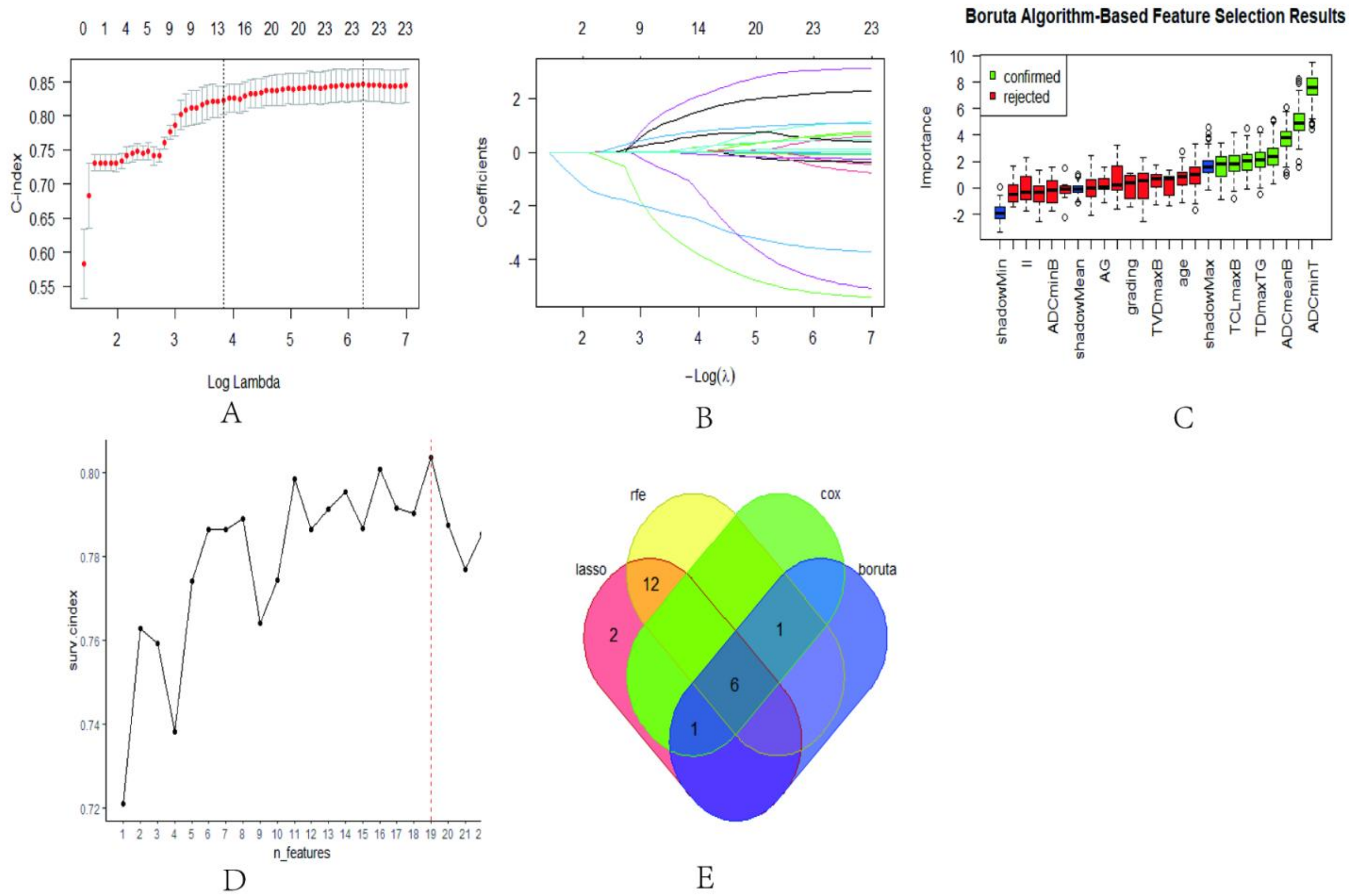

Fig.S1 Supplementary Material

A: C-index vs. log(lambda) in LASSO regression.

B:LASSO coefficient profile plot.

C: Boruta algorithm feature selection results.

D: RFE feature selection curve (C-index vs. number of features).

E: Venn diagram of overlapping features from LASSO, RFE, COX, and Boruta.

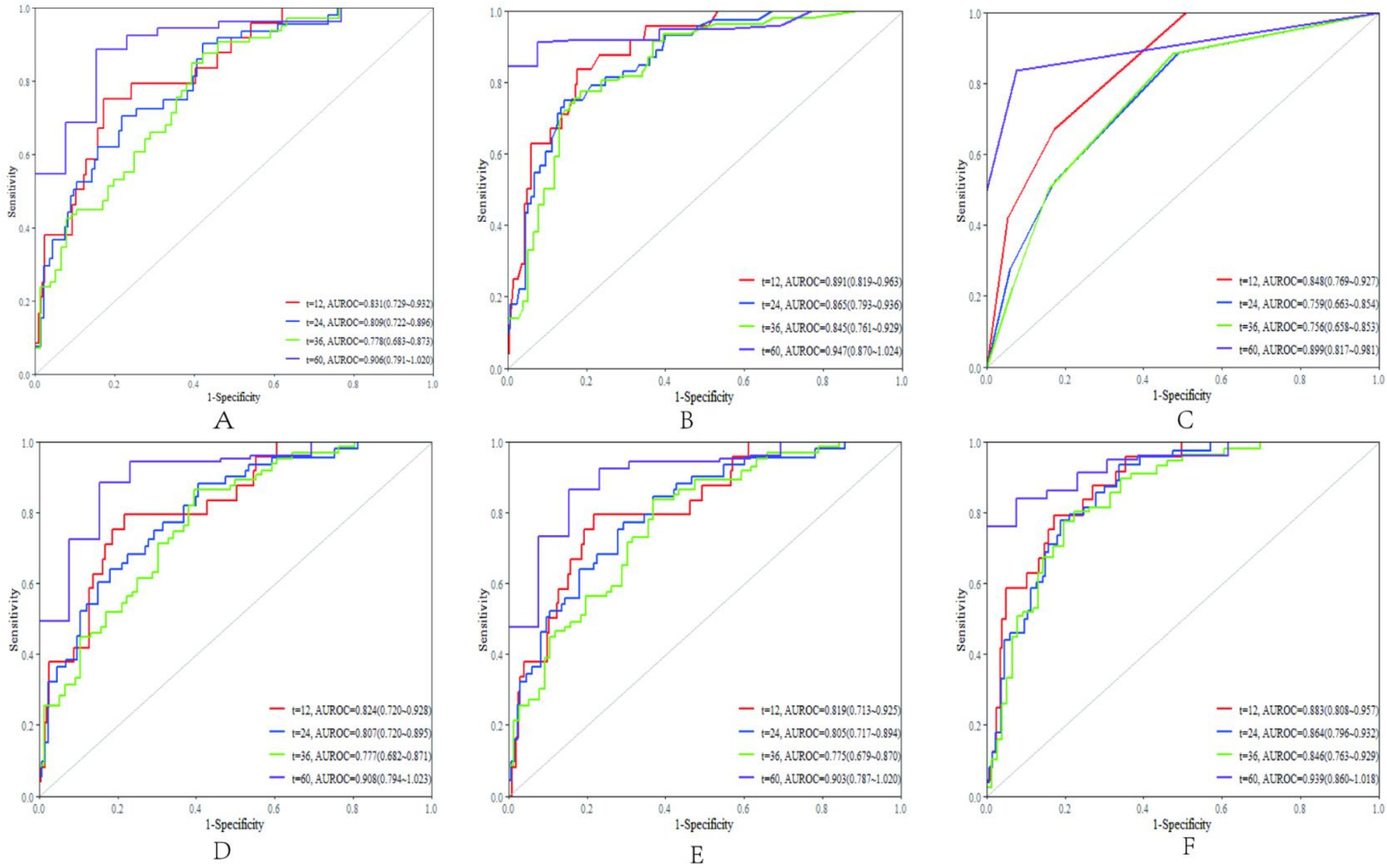

Fig.S2 Supplementary Material

A:ROC curve of the coxph model in the training set

B:ROC curve of the gbm model in the training set

C:ROC curve of the ctree model in the training set

D:ROC curve of the ridge model in the training set

E:ROC curve of the enet model in the training set

F:ROC curve of the rsf model in the training set

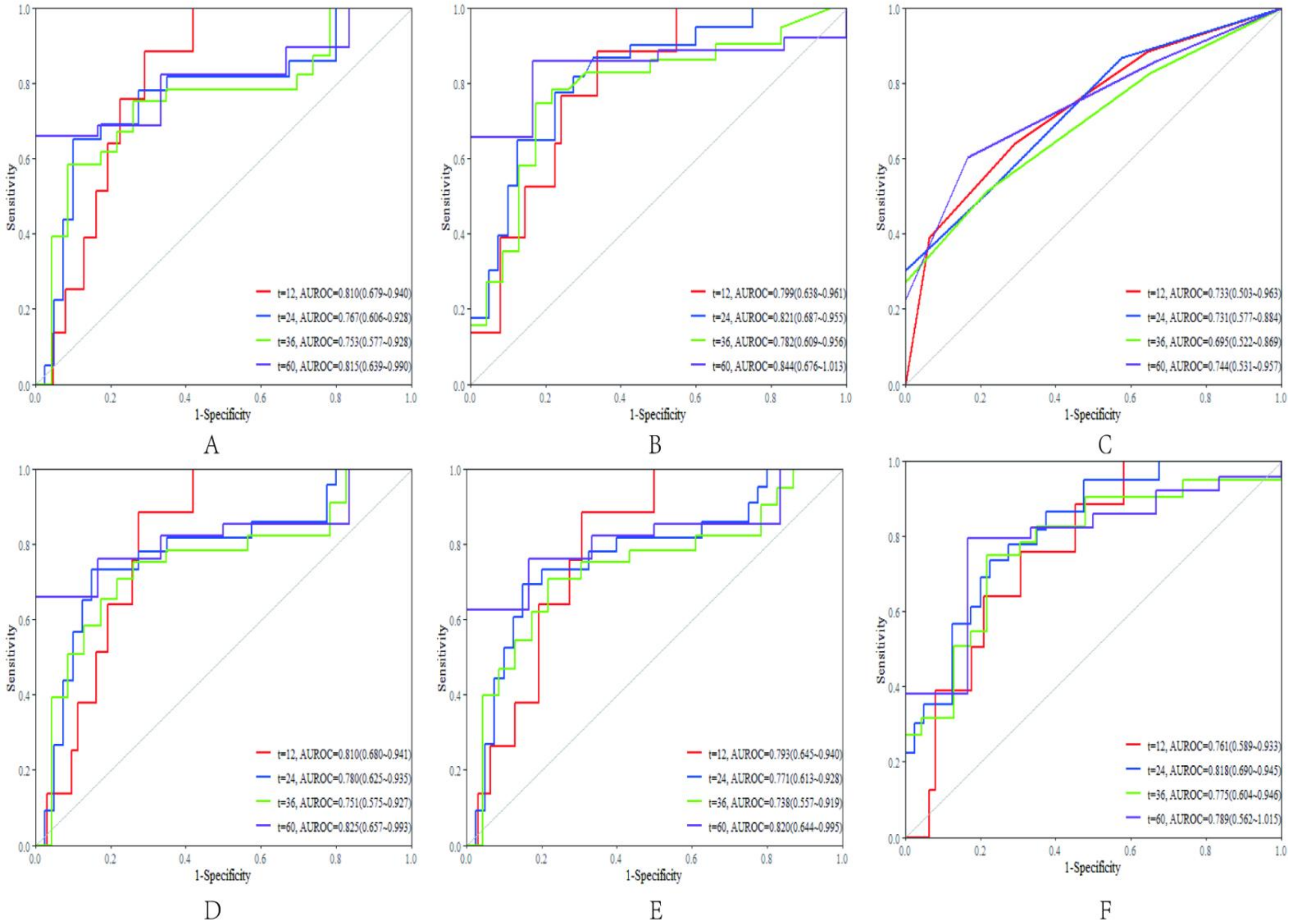

Fig.S3 Supplementary Material

A:ROC curve of the coxph model in the test set

B:ROC curve of the gbm model in the test set

C:ROC curve of the ctree model in the test set

D:ROC curve of the ridge model in the test set

E:ROC curve of the enet model in the test set

F:ROC curve of the rsf model in the test set

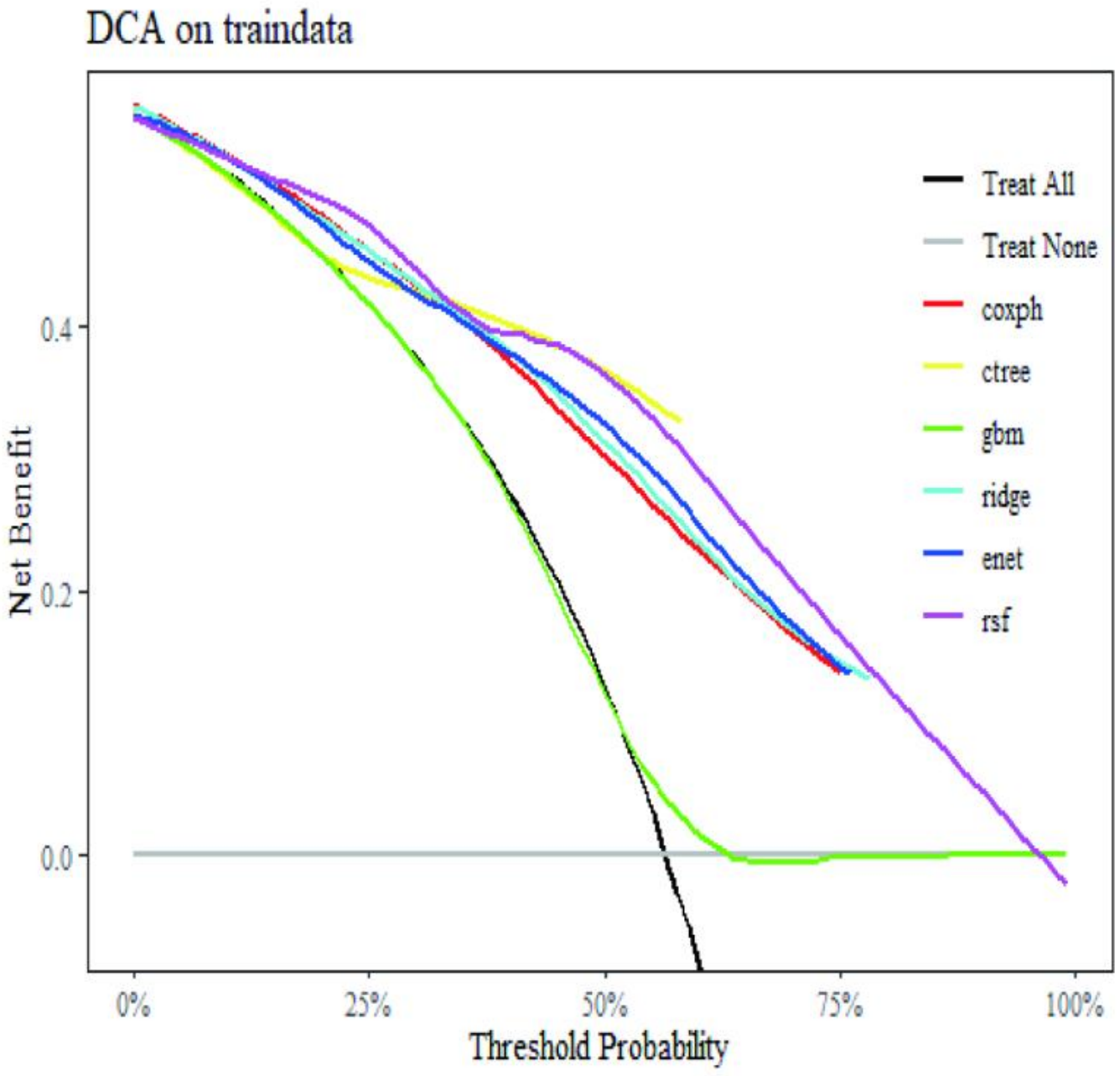

A

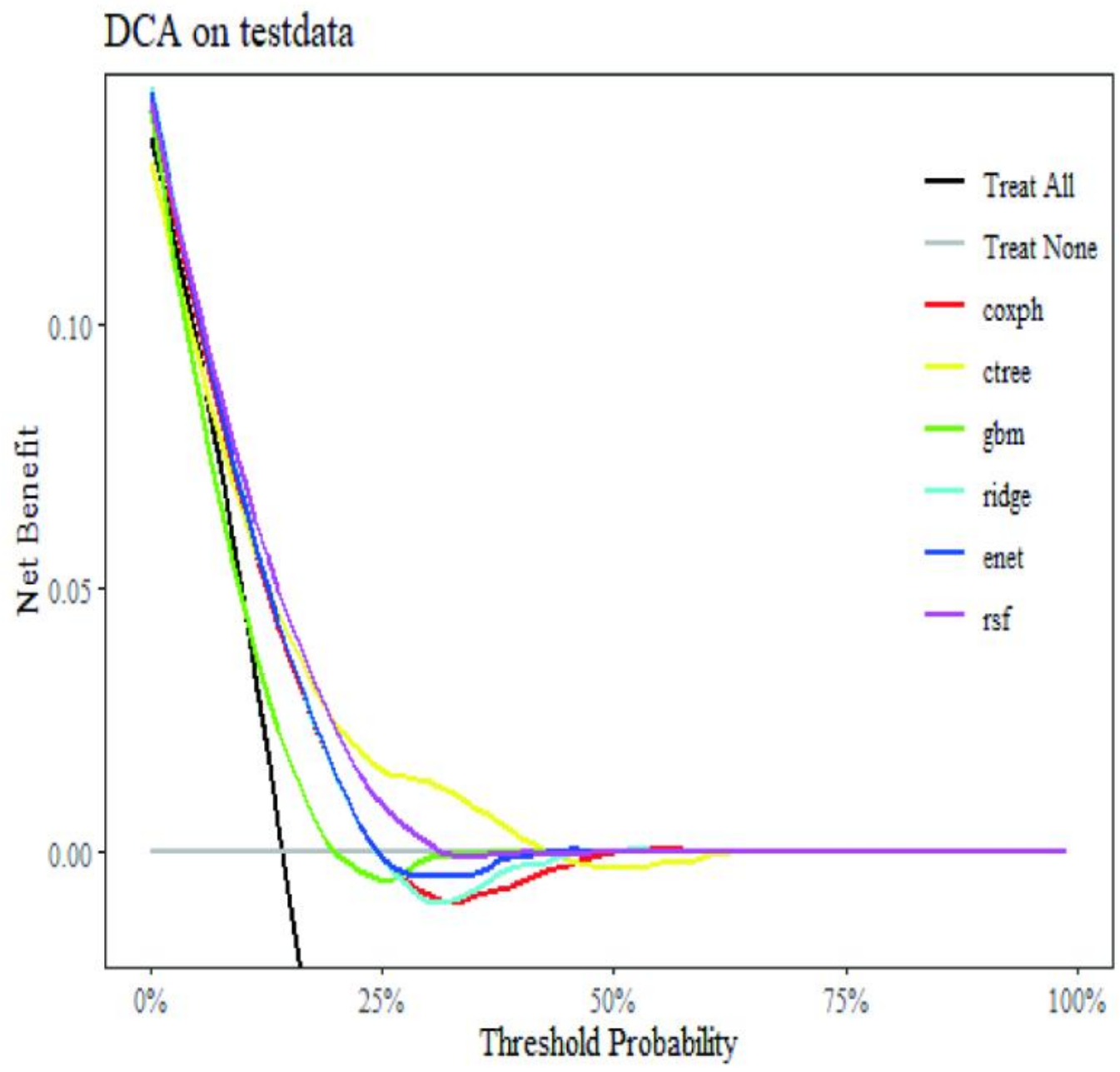

B

Fig.S4 Supplementary Material

A:Decision curve analysis (DCA) of different prediction models in the training set.

B:Decision curve analysis (DCA) of different prediction models in the test set.

Supplementary Table S1

Hyperparameter grid search ranges for six survival models optimized via 5-fold cross-validation within training cohort only

Model Type	Tuned Hyperparameters	Grid Search Candidate Values (mlr3 grid_search resolution = 3 for numeric tuning spaces)
Cox proportional hazards (coxph)	No penalized hyperparameters; feature subset fixed after multi-algorithm feature selection	N/A (fixed predictor set only)
Ridge Cox regression	Regularization penalty λ	0.001, 0.01, 0.1, 1, 5, 10, 50, 100
Elastic net Cox regression	Regularization penalty λ; mixing parameter α (L1/L2 ratio)	λ: 0.001, 0.01, 0.1, 1, 5, 10, 50, 100α: 0.1, 0.5, 0.9
Conditional Inference Tree (CTree)	Minimum node size; split significance threshold p-value	Min node size: 5, 12, 20Split p-threshold: 0.01, 0.05, 0.1
Gradient Boosting Machine (GBM)	Learning rate; max tree depth; subsample fraction; number of trees	Learning rate: 0.01, 0.05, 0.1Max depth: 2, 3, 4Subsample: 0.6, 0.75, 0.9N trees: 50, 100, 200
Random Survival Forest (RSF, randomForestSRC::rfsrc)	ntree (number of trees); mtry (variables per split); nodesize (terminal node minimum sample size); splitrule	ntree: 200, 350, 500mtry: 3, 4, 5nodesize: 15, 18, 21splitrule: logrank (fixed, not tuned)

Table Footnote

1. All grid search procedures were executed exclusively on the training cohort (n=256) via 5-fold stratified internal cross-validation stratified by recurrence event status; the hyperparameter combination yielding the maximal cross-validation C-index was retained for final model construction.
2. No validation set data was accessed during any hyperparameter tuning or feature screening steps to eliminate data leakage.
3. For RSF, hyperparameter spaces were defined via mlr3::to_tune() with tuner = tnr("grid_search", resolution = 3) to generate evenly spaced candidate values between the specified lower and upper bounds, consistent with the provided R analytical pipeline.
4. Split rule for RSF was fixed as logrank (default splitting criterion for survival forest) and excluded from tuning space following standard randomForestSRC workflow.

Supplementary Table 2.

Number of patients at risk and censored observations at each time point for time-dependent ROC analysis

Time point	Patients at risk	Censored patients
12 months	281	202
24 months	175	136
36 months	104	84
60 months	22	22

Supplementary Table S2 Notes：

Patients at risk at each time point were defined as recurrence-free patients with follow-up longer than the corresponding month threshold. A total of 72 patients had follow-up ≥60 months in the whole cohort, among whom only 22 remained recurrence-free and constituted the risk set at 60 months.
